# Diagnosis and Management of Monostotic Fibrous Dysplasia of the Tibia in an Adolescent Patient: A Case Report

**DOI:** 10.7759/cureus.56052

**Published:** 2024-03-12

**Authors:** Ashutosh Lohiya, Nareshkumar Dhaniwala, Sanjay V Deshpande, Vivek H Jadawala, Saksham Goyal, Suhit Naseri

**Affiliations:** 1 Department of Orthopaedics, Jawaharlal Nehru Medical College, Datta Meghe Institute of Higher Education and Research, Wardha, IND; 2 Department of Pathology, Jawaharlal Nehru Medical College, Datta Meghe Institute of Higher Education and Research, Wardha, IND

**Keywords:** tibia, benign bone tumor, synthetic bone graft, fibrous dysplasia, monostotic

## Abstract

A rare benign bone condition called monostotic fibrous dysplasia (MFD) is characterized by the growth of fibrous tissue in place of a normal bone. It may lead to deformity in the affected bone, pain, and a pathologic fracture due to bone weakness. Hereunder, a case report of MFD in a 17-year-old male adolescent presenting to the hospital with localized bone pain and swelling in his right tibia is presented. After clinical examination and radiographic imaging, a provisional diagnosis of benign osteolytic lesion was considered. A magnetic resonance imaging (MRI) scan of the leg suggested the possibility of fibrous dysplasia or adamantinoma. The patient was managed with an intralesional curettage of the dysplastic bone and packing the cavity with blocks of a synthetic bone. The excised material was sent for histopathology, which established the diagnosis of fibrous dysplasia.

## Introduction

Monostotic fibrous dysplasia (MFD) is a benign bone tumor that can occur at any age, but it most commonly presents in childhood and adolescence [1]. It accounts for 10% of all bone tumors and approximately 70% of all cases of fibrous dysplasia [2]. This condition is characterized by the replacement of normal bone and marrow by fibrous tissue and woven bone [3]. Patients typically present with pain, deformity, limb length discrepancy, and pathologic fractures [4]. The lesions are monostotic when only one bone is involved, compared to polyostotic fibrous dysplasia that affects multiple bones. The most commonly affected bones are the ribs, proximal femur, tibia, and craniofacial bones [5]. While the exact pathogenesis is unclear, MFD is thought to be caused by somatic activating mutations in the guanine nucleotide-binding protein, alpha-stimulating activity polypeptide (GNAS) gene, which leads to increased proliferation and differentiation of bone marrow stromal cells [6]. Radiographs demonstrate a well-demarcated lytic lesion with a ground-glass appearance and thinning of the surrounding cortex [2]. CT and MRI scans can further characterize the lesion and help detect cortical irregularities [7]. A definitive diagnosis requires a biopsy, which shows spindle-shaped fibroblasts in a matrix of trabeculated woven bone and irregular calcification [3]. Treatment is aimed at managing symptoms and preventing progression and complications, such as fractures. Bisphosphonates are often used for pain relief [8]. Surgery is considered for the stabilization and correction of deformities in weight-bearing bones and joints [9].

## Case presentation

**Figure 1:**
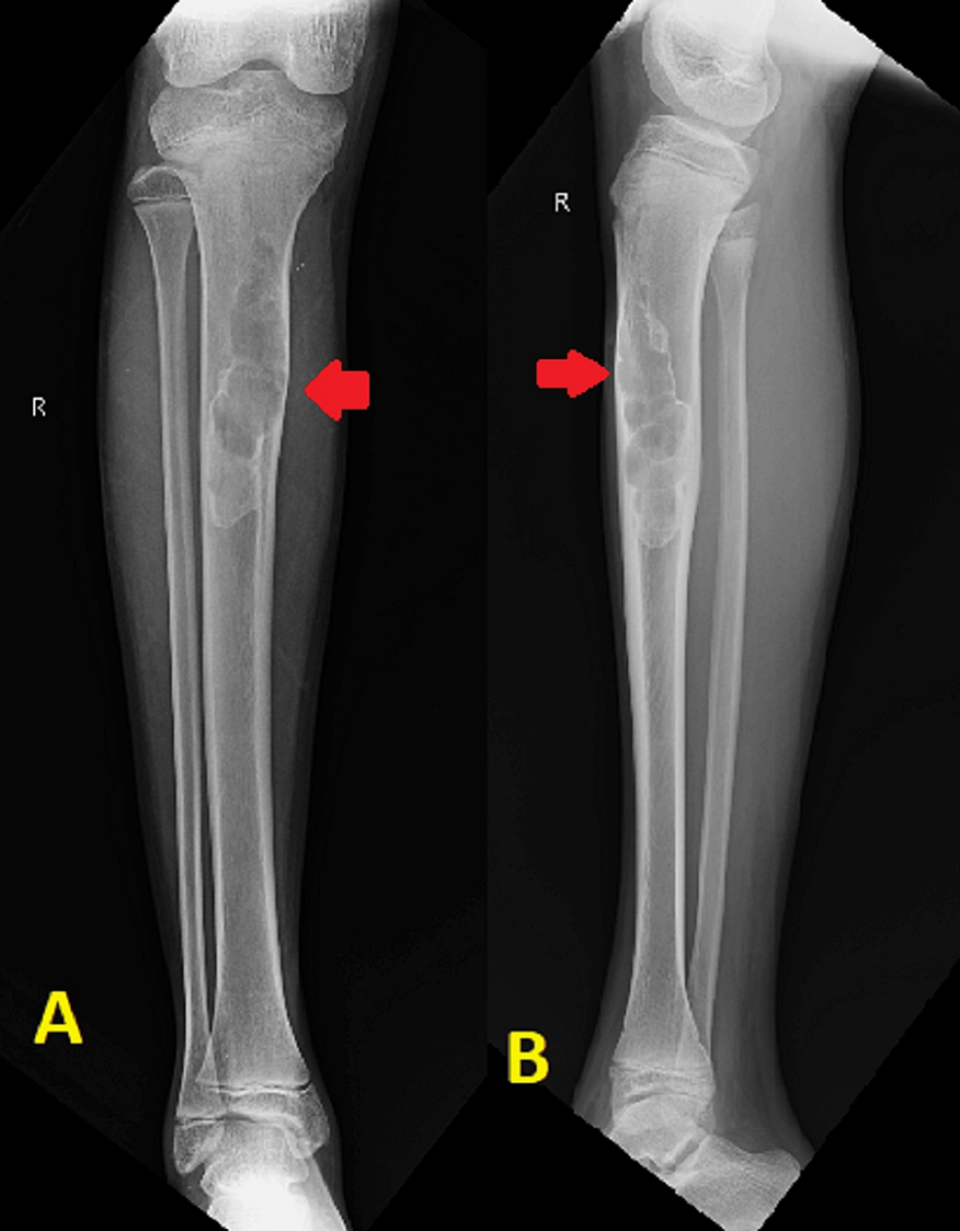
X-ray of the right leg in anteroposterior (A) and lateral (B) views showing an osteolytic lesion in proximal one-third to the midshaft tibia.

A male patient, 17 years old, presented to the orthopedic department of a tertiary care hospital in central India complaining of progressive right leg pain and swelling for the past six months. The pain was localized only to the anterior aspect of the leg and was aggravated by weight-bearing activities, like walking, running, and jumping. There was no history of trauma, falls, or any other symptoms, such as fever, night sweats, or unintentional weight loss. The patient's medical history was unremarkable, with no significant past illnesses or surgeries. On physical examination, there was tenderness and bony swelling over the mid-shaft of the right tibia. The swelling was localized on the anteromedial aspect of the right tibia starting 6 cm from the joint line and extending about 8 cm distally. It was hard in consistency and the tibial surface appeared irregular in feel. The tibia was enlarged mediolaterally at the site of swelling. There were no local signs of inflammation, the skin was freely mobile, and the range of movement of the right knee and ankle joints was within normal limits. There was no evidence of ligamentous laxity or neurovascular deficit. Systemic and general examinations revealed no abnormalities. An osteolytic lesion with a ground-glass appearance involving the proximal third to the mid-shaft region of the right tibia was discovered during the initial radiographic evaluation using anteroposterior and lateral views. The lesion was multiloculated and irregular in shape causing thinning of the anterior and medial cortex (Figure 1).

**Figure 2:**
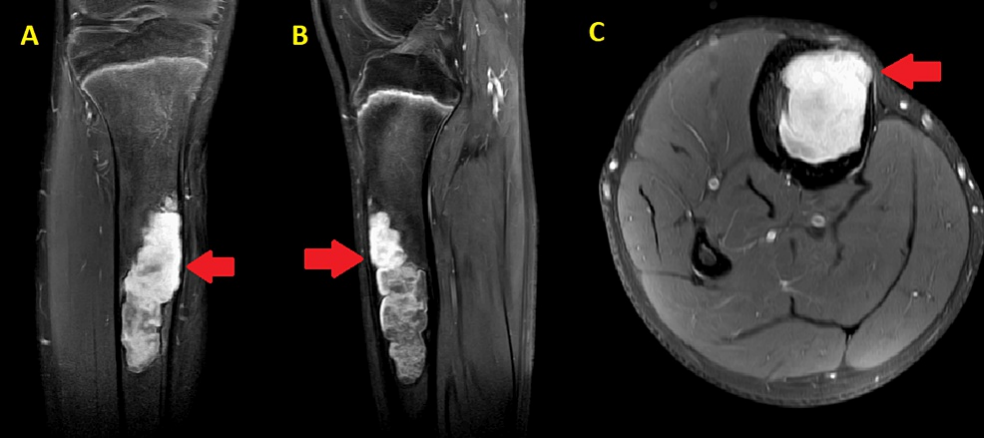
MRI in contrast study right leg in coronal (A), sagittal (B), and axial (C) T1 views shows a well-defined diaphyseal lesion of size 10.3 x 2.8 cm in the tibia with a narrow zone of transition.

There was no evidence of any pathological fracture or periosteal reaction. Further characterization of the lesion was done through magnetic resonance imaging (MRI), which demonstrated a well-defined diaphyseal lesion of size 10.3 x 2.8 cm in the tibia with a narrow zone of transition. These findings suggested the possibility of adamantinoma or fibrous dysplasia. Cortical thinning and expansion of the affected bone as seen on MRI are consistent with fibrous dysplasia. MRI revealed no evidence of surrounding soft tissue invasion or neurovascular compromise. Based on these findings, a provisional diagnosis of MFD of the right tibia was considered (Figure 2).

**Figure 3:**
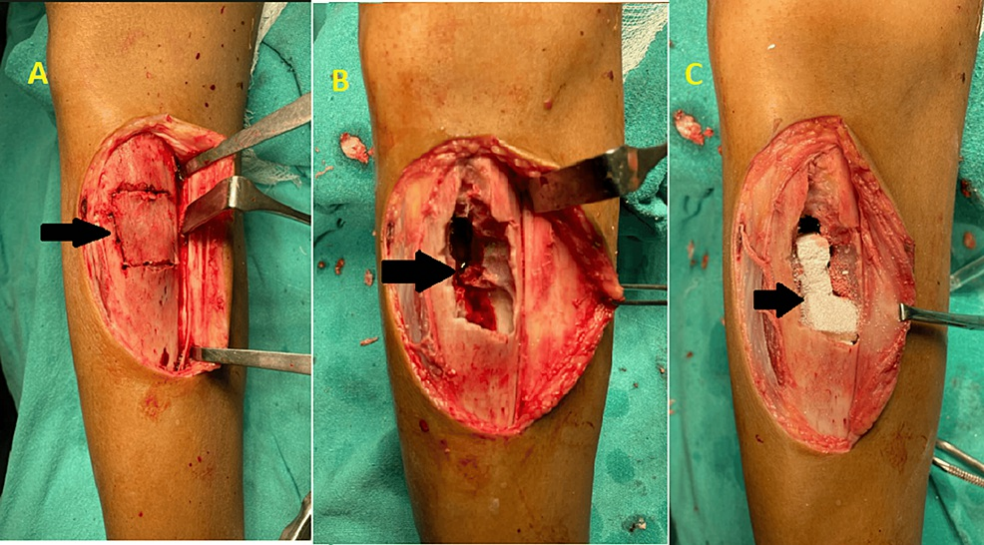
Intraoperative steps showing (A) outline of the area from where the curettage is to be done, (B) cavity formed after curettage of the lesion, and (C) synthetic bone substitute filled in the cavity.

Given the symptomatic nature of the lesion and the risk of progression, surgical intervention was recommended. The patient underwent surgery under spinal anesthesia and tourniquet application. He underwent excision of the fibrous dysplastic lesion using thorough intralesional curettage, followed by packing of the area using a synthetic bone graft substitute (G bone), to promote bone healing, stability, and strength. Figure 3 shows the intraoperative steps of marking the area, curettage, and packing the curetted cavity with a synthetic bone graft substitute. During curettage, continuity of the marrow cavity proximally and distally was established for better vascularity. The closure was done in layers, and antibiotics were used for three days. Stitches were removed on the 10th postoperative day, and partial weight bearing was permitted two weeks after surgery.

**Figure 4:**
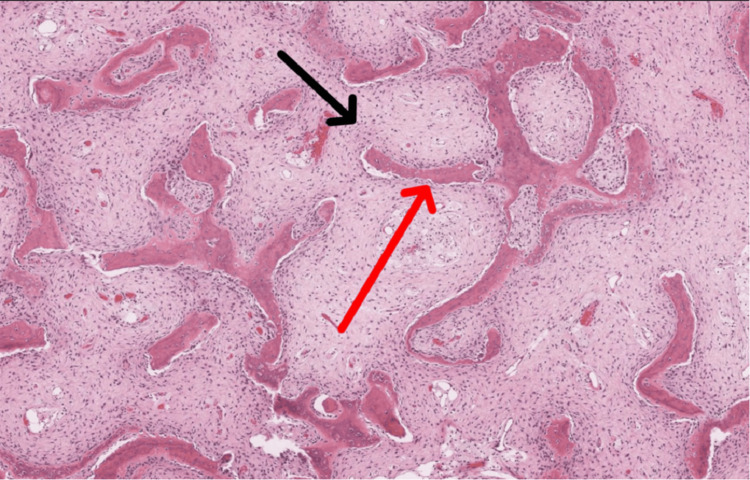
Histopathology of the bony lesion. Fibrous tissue composed of spindled cells with tapered nuclei (black arrow) and bone trabeculae composed of curvilinear woven bone, appearing to arise from fibrous tissue (red arrow).

The synthetic bone graft substitute (G bone) was used because it has excellent osteoconductive and to some extent osteoinductive properties. The curetted material was whitish brown in color and firm in consistency, suggestive of fibrous tissue within the lesion. The collected material was sent for histopathology, which showed fibrous tissue composed of spindle cells with tapered nuclei. It also showed bone trabeculae composed of curvilinear woven bone arising from fibrous tissue (Figure 4).

**Figure 5:**
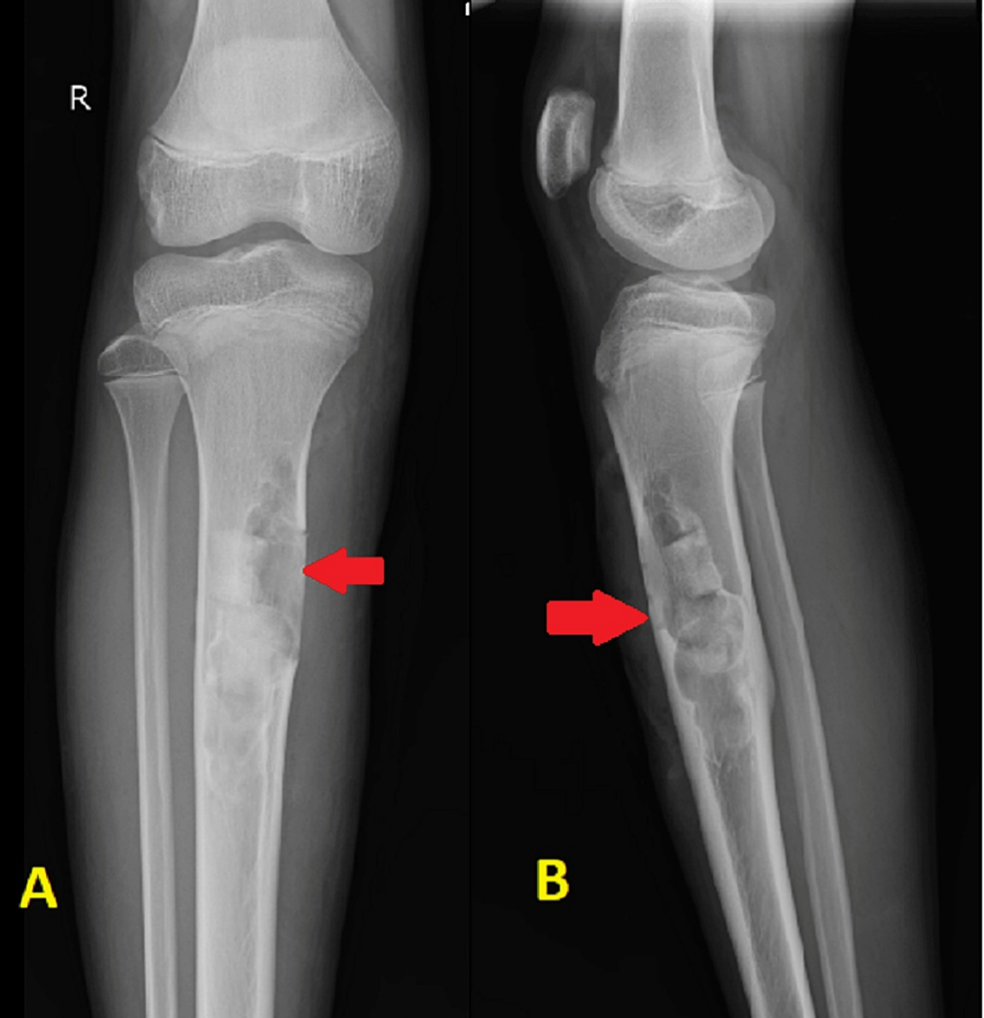
Postoperative X-ray of the right leg in anteroposterior (A) and lateral (B) views, showing the synthetic bone substitute post curettage.

These findings established the diagnosis of MFD of the right tibia in the present case. Postoperatively, the patient experienced gradual resolution of pain and swelling over the right tibia (Figure 5). He was instructed to adhere to a progressive weight-bearing protocol and initiated physical therapy to optimize functional recovery and prevent complications, such as stiffness or muscle atrophy. Postoperative radiographic evaluation showed evidence of bone healing and integration of the G bone graft. At six months postoperatively, the patient was able to fully bear weight without pain and had returned to his pre-disease level of physical activity. At 18 months follow-up, the patient continued to be asymptomatic without any evidence of recurrence or limitation of activity.

## Discussion

MFD is a benign skeletal disorder characterized by the replacement of a normal bone with fibrous tissue, leading to bone deformity and structural weakness. In this case, a 17-year-old male presented with localized pain and swelling in the right tibia, ultimately diagnosed as MFD. The most prevalent types of fibrous dysplasia are single-bone lesions with a monostotic appearance that do not exhibit any other disruption [10]. Monostotic fibrous dysplasias are known to expand proportionately until skeletal growth. Less frequently occurring polyostotic forms frequently continue to grow even after full skeletal maturation. In polyostotic fibrous dysplasia, this characteristic may lead to increased deformity and a higher incidence of pathologic fractures [2]. Shepherd's crook deformity of the proximal section of the femur, a disparity in the lengths of bilateral limbs, is the most prevalent skeletal malformation in fibrous dysplasia [11]. A peripheral sclerotic bone lesion that is well-marginalized and has several patterns as seen on AP and lateral X-rays of the part is indicative of fibrous dysplasia.

The pattern of the lesion depends on the amount of fibrous components, calcification, and bony trabeculae. These patterns may appear ground glass-like, lucent, sclerotic, or mixed [12]. These lesions do not have a cortical disturbance or periosteal reaction, but there may be focal cortical thinning and endosteal scalloping. When radiographic characteristics suggest fibrous dysplasia, it can be differentiated from central osteosarcomas of lower grade using MRI. Most fibrous dysplasia is isointense, primarily consisting of skeletal muscle on T1-weighted images, according to a visual evaluation by Saha and colleagues [13]. Lesions are usually heterogeneously hyperintense on T2-weighted images, with patches within the lesion that are hypo-, iso-, or noticeably hyperintense. Fibrous dysplasia may manifest as homogeneous enhancement, rim enhancement, patchy center enhancement, or any combination of these on an enhanced MRI scan. The immature, shaped like a spindle, fibroblast-like cells in the bone marrow that produce fibrous tissue that extends from the medullary cavity into the cortical bone are the histopathologic hallmark of fibrous dysplasia [14]. Some have compared these immature oddly formed trabeculae to a "soup of alphabets" or "Chinese characters."

## Conclusions

This case highlights the successful management of MFD in an adolescent patient through surgical excision and G-bone insertion. The comprehensive diagnostic approach, including clinical evaluation and advanced imaging modalities, facilitated accurate diagnosis and guided appropriate treatment planning. Surgical intervention effectively alleviated symptoms and restored normal function, emphasizing the importance of tailored management strategies in cases of symptomatic MFD. Long-term follow-up is crucial to monitor for recurrence or graft-related complications and ensure continued patient well-being. Further research is warranted to explore alternative treatment modalities and optimize outcomes for patients with MFD.
